# An *Ab Initio* Electronic Structure Investigation of the Ground and Excited States of ScH^+^, YH^+^, and LaH^+^

**DOI:** 10.3390/molecules30112435

**Published:** 2025-06-02

**Authors:** Isuru R. Ariyarathna

**Affiliations:** Los Alamos National Laboratory, Theoretical Division, Los Alamos, NM 87545, USA; isuru@lanl.gov

**Keywords:** MRCI, CCSD(T), fcFCI, CBS-fcFCI-δcore-δSO, electronic structures, excited states

## Abstract

Multireference configuration interaction (MRCI), Davidson-corrected MRCI (MRCI+Q), coupled-cluster singles, doubles, and perturbative triples [CCSD(T)], and frozen-core full configuration interaction (fcFCI) calculations were carried out using large, correlation-consistent basis sets to investigate the excited states of the Sc atom and the spin–free and spin–orbit coupled potential energy profiles, energetics, spectroscopic constants, and electron populations of low-lying states of MH^+^ (M = Sc, Y, La). The core electron correlation effects, complete basis set effects, and spin–orbit coupling effects were also evaluated. The first four electronic states of all MH^+^ are 1^2^Δ, 1^2^Σ^+^, 1^2^Π, and 2^2^Σ^+^ with 1σ^2^1δ^1^, 1σ^2^2σ^1^, 1σ^2^1π^1^, and 1σ^2^3σ^1^ single-reference electron configurations, respectively. These states of MH^+^ can be represented by the M^2+^H^–^ ionic structure. The ground states of ScH^+^, YH^+^, and LaH^+^ are 1^2^Δ_3/2_, 1^2^Σ^+^_1/2_, and 1^2^Δ_3/2_ with 55.45, 60.54, and 62.34 kcal/mol bond energies, respectively. The core electron correlation was found to be vital for gaining accurate predictions on the ground and excited state properties of MH^+^. The spin–orbit coupling effects are minor for ScH^+^ but become substantial moving to YH^+^ and LaH^+^. Overall, the results of this work are in good agreement with the limited set of experimental findings of MH^+^ available in the literature and will be of use for future investigations. Furthermore, the theoretical approaches, findings, and trends reported here are expected to aid studies of similar species.

## 1. Introduction

Electronic structure investigations of molecular complexes are vital for understanding their underlying chemicophysical properties and subsequently optimizing their applications in a variety of industrial processes. Especially, many correlated metal-based species display singular catalytic, electronic, magnetic, and photochemical properties arising from their intricate electron arrangements [1,2,3,4,5,6,7,8]. Therefore, over the years, many high-level quantum chemical investigations have been made to explore the electronic structures and properties of metal-based diatomic constituents aiming to comprehend the chemistries of their large complexes. Furthermore, such theoretical studies of diatomic species are necessary to interpret their experimental spectra and produce models to understand their chemistries under a variety of conditions. In this area of research, the studies of neutral and charged transition metal- and actinide-based metal hydrides are dominant due to their applications in the fields of astrophysics, catalysis, and renewable energy [7,9,10,11,12]. Surprisingly, such investigations on early transition metal (Sc and Y) and lanthanide (La) monohydride cations are scarce in the literature, and their chemical bonding and spectroscopic properties are poorly understood. Hence, in the present work, we have adopted several high-level *ab initio* methods to explore the electronic structures of ground and excited states of MH^+^ (M = Sc, Y, La), aiming to assist their future investigations.

To the best of our knowledge, the first experimental chemical bonding study of ScH^+^ goes back to the work by Tolbert and Beauchamp in 1984 [13]. They measured the bond energy of ScH^+^ to be 54 ± 4 kcal/mol by conducting an ion beam experiment [13]. A year later, Alvarado-Swaisgood and Harrison [14] reported a dissociation energy (D_0_) of 52.7 kcal/mol for ScH^+^ which is within the range of uncertainty of the experimental D_0_ of Tolbert and Beauchamp [13] by performing multi-configurational self-consistent field (MCSCF) quantum chemical calculations. Furthermore, this theoretical study assigned a ^2^Δ term symbol for the ground state of ScH^+^ and provided evidence for two closely lying ^2^Π and ^2^Σ^+^ excited states [14]. In 1986, Elkind and Armentrout measured the D_0_ of ScH^+^ using a guided ion beam mass spectrometric experiment and reported a value of 55.3 ± 2 kcal/mol [15] which agreed well with the previous D_0_ values by Tolbert and Beauchamp [13] and Alvarado-Swaisgood and Harrison [14]. They further reported a D_0_ of 58 ± 3 kcal/mol for YH^+^ [15]. In 1987, Pettersson et al. [16], reported theoretical D_0_, bond length (r_e_), and harmonic vibrational frequency (ω_e_) values of ScH^+^(^2^Δ) (i.e., 56.0 kcal/mol, 1.830 Å, and 1568 cm^−1^, respectively) and adiabatic excitation energy (T_e_), r_e_, and ω_e_ of ScH^+^(^2^Σ^+^) (i.e., 1700 cm^−1^, 1.788 Å, and 1571 cm^−1^, respectively) under the modified coupled pair functional (MCPF) theory. Furthermore, using the same method, they assigned a ^2^Σ^+^ ground state and a 59.4 kcal/mol D_0_ for YH^+^ [16], which is in agreement with its experimental D_0_ by Elkind and Armentrout [15]. Two years later, Armentrout’s group carried out further ion beam mass spectrometric studies on ScH^+^ and YH^+^ and re-reported their D_0_ values to be 56.27 ± 2.08 and 61.34 ± 1.38 kcal/mol, respectively [17]. Under the same experimental approach, they measured the D_0_ of LaH^+^ to be 57.19 ± 2.08 kcal/mol [17]. Soon after, Das and Balasubramanian reported a second-order configuration interaction (SOCI) theoretical study for LaH^+^ and predicted a D_e_ (zero-point energy unaccounted bond energy) of 58.57 kcal/mol for its ^2^Δ ground state [18]. Their calculations further predicted the first two excited electronic states of LaH^+^ (i.e., ^2^Σ^+^ and ^2^Π) to be at 1948 and 2855 cm^−1^ [18].

Even though the bond energies of the ground states and some spectroscopic parameters of a few states of MH^+^ are available in the literature, these species are lacking a thorough analysis under the state-of-the-art wave function theories conjoined with large basis sets. For example, high-level MRCI (≡ MRCISD) calculations are often being performed with correlation-consistent basis sets to investigate correlated transition metal-, lanthanide-, and actinide-based diatomic species. Moreover, the MRCI coefficients and correlation energies can be used to evaluate the approximate quadruple substitution effect (+Q), hence often MRCI+Q findings are more accurate and reliable compared to the MRCI findings. A thorough analysis based on CCSD(T) which addresses the perturbative triples contribution on top of the singles and doubles electron correlation effects and fcFCI (in MH^+^ cases, fcFCI accounts for single, double, and triple electron correlation effects) is also absent for MH^+^ species. Hence, in the present work, the aforementioned high-level theories were used to provide a comprehensive analysis of the electronic structures, energetics, and spectroscopic parameters of MH^+^ species. Specifically, we have introduced full potential energy profiles of spin–free and spin–orbit curves of MH^+^ at the MRCI level with correlation-consistent basis sets to determine their spectroscopic properties and bond dissociations. Then, we have adopted highly accurate single-reference CCSD(T) and fcFCI levels of theory to further investigate energy related properties and spectroscopic constants of MH^+^. The core electron correlation effects, complete basis set effects, and spin–orbit coupling effects on their properties were also examined.

## 2. Results and Discussion

### 2.1. Sc Atom

As the first step of this work, the Sc atom was studied aiming to test the accuracy of three multireference approaches on its excited states. Specifically, the MRCI/aug-cc-pV5Z-DK (MRCI_Sc_), MRCI/aug-cc-pwCV5Z-DK (C-MRCI_Sc_), and MRCI+Q/aug-cc-pwCV5Z-DK (C-MRCI+Q_Sc_) calculations were performed to obtain the excitation energies of all *J* states of the first six electronic states of Sc (Table 1). The ground electronic state of the Sc atom is a ^2^D ([Ar]3d^1^4s^2^) and its two *J* terms (i.e., *J* = 3/2 and 5/2) are energetically separated by 168 cm^−1^ [19]. In agreement with the experiment, all our theoretical approaches predicted a ^2^D_3/2_ ground state for the Sc atom (Table 1). The MRCI_Sc_ and C-MRCI_Sc_ predicted excitation energies for the ^2^D_5/2_ are in good agreement with the experiment (i.e., 182, 193, and 168 cm^−1^, respectively). Among the three approaches, C-MRCI+Q_Sc_ predicted the highest ^2^D_3/2_ → ^2^D_5/2_ excitation energy (i.e., 221 cm^−1^). According to experiment, the first two excited electronic states of the Sc atom (i.e., ^4^F and ^2^F) lie at 11,520–11,678 and 14,926–15,042 cm^−1^, respectively [19]. All our calculations overestimated the excitation energies of the *J* = 3/2, 5/2, 7/2, and 9/2 of ^4^F and *J* = 5/2 and 7/2 of ^2^F, similar to the excitation energy of ^2^D_5/2_ (Table 1). Specifically, the largest discrepancies with respect to the experiment (for the *J* states of ^4^F and ^2^F) were provided by the MRCI_Sc_ (~2850 cm^−1^), whereas the overestimations by both C-MRCI_Sc_ and C-MRCI+Q_Sc_ levels are ~700–800 cm^−1^. Both MRCI_Sc_ and C-MRCI_Sc_ underestimated the excitation energies of ^4^F^o^ by ~1100 and ~300 cm^−1^ while the corresponding C-MRCI+Q_Sc_ excitation energies are in reasonable agreement with the experiment (the discrepancies are only 74–90 cm^−1^). Similarly, the MRCI_Sc_ substantially underestimated the excitation energies of the next two excited electronic states, ^4^D° and ^2^D°, of the Sc atom (by 1173–1413 cm^−1^). The excitation energies predicted by both C-MRCI_Sc_ and C-MRCI+Q_Sc_ for these two states only differ by 9–172 cm^−1^ with respect to the experiment. Overall, both C-MRCI_Sc_ and C-MRCI+Q_Sc_ representations of the excited states of the Sc atom are significantly better compared to the representation of MRCI_Sc_, which highlights the importance of the core electron correlation for obtaining accurate excitation energies. Furthermore, the +Q correction was found to improve the predictions of the higher excited states of the Sc atom. Specifically, the C-MRCI+Q excitation energies of the Sc atom for all *J* states of ^2^F, ^4^F^o^, ^4^D°, and ^2^D° (except for the *J* = 3/2 and *J* = 5/2 of ^4^D°) are in better agreement with the experiment compared to the C-MRCI (Table 1).

### 2.2. ScH^+^

**Figure 1:**
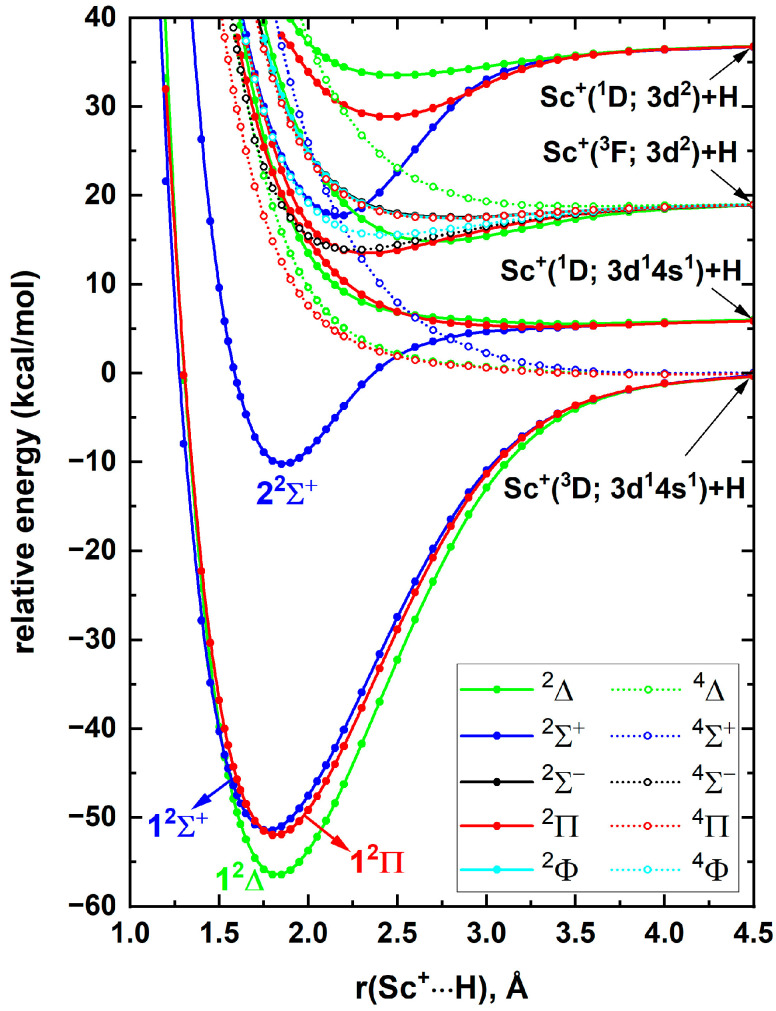
A5Z-MRCI PECs of ScH^+^ as a function of Sc^+^···H distance [r(Sc^+^···H), Å]. The relative energies are referenced to the Sc^+^(^3^D) + H(^2^S) fragments placed at 200 Å, which is set to 0 kcal/mol. In all reactants the H atom is in its ground ^2^S state.

To study ScH^+^, the reactions between a few low-lying electronic states of Sc^+^ and the ground state of H atom were considered. Specifically, the Sc^+^(^3^D; 3d^1^4s^1^)+H(^2^S), Sc^+^(^1^D; 3d^1^4s^1^)+H(^2^S), Sc^+^(^3^F; 3d^2^)+H(^2^S), and Sc^+^(^1^D; 3d^2^)+H(^2^S) interactions were investigated. These combinations produce ^4,2^[Σ^+^, Π, Δ], ^2^[Σ^+^, Π, Δ], ^4,2^[Σ^–^, Π, Δ, Φ], and ^2^[Σ^+^, Π, Δ] molecular states of ScH^+^, respectively, and their PECs are given in Figure 1. All the quartet-spin PECs resulting from the ground state fragments [i.e., Sc^+^(^3^D; 3d^1^4s^1^)+H(^2^S)] are repulsive in nature. On the other hand, the doublet-spin PECs of the same reactants are strongly attractive and create the first three electronic states of ScH^+^ (i.e., 1^2^Δ, 1^2^Σ^+^, and 1^2^Π). According to Figure 1, the first two excited states of ScH^+^ (i.e., 1^2^Σ^+^ and 1^2^Π) lie ~4 kcal/mol above the 1^2^Δ ground state. Recall that the core electron correlation significantly improved the excitation energies of the Sc atom (Section 2.1) and similarly the higher electron correlation effects may be necessary for identifying the exact ordering of the closely lying 1^2^Σ^+^ and 1^2^Π states of ScH^+^. Indeed, we discuss such electron correlation effects on the excited states and a few other properties of ScH^+^ in detail later in this section. The Sc^+^(^1^D; 3d^1^4s^1^)+H(^2^S) interaction produces only one stable potential energy minimum (i.e., 2^2^Σ^+^), which is the third excited state of ScH^+^. The Sc^+^(^3^F; 3d^2^)+H(^2^S) interaction does not produce strongly attractive PECs except for a few PECs with shallow minima (i.e., ^2^Π, ^4^Σ^-^, ^2^Δ, and ^4^Φ) around 2.2–2.7 Å.

**Figure 2:**
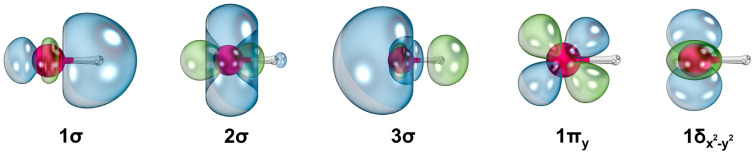
Select CASSCF state average molecular orbitals of ScH^+^. The Sc and H atoms of each orbital plot are shown in magenta and gray spheres, respectively. The two phases of orbitals are given in blue and green. The rotations of 1π_y_ and $1\delta_{x^{2}-y^{2}}$ orbitals by 90^o^ and 45^o^ along the z-axis (Sc–H bond) produce the contours of 1π_x_ and 1δ_xy_, respectively. A threshold of 90% was used to plot the contours. IboView [20] was used to produce molecular orbitals.

The first four electronic states of ScH^+^ (i.e., 1^2^Δ, 1^2^Σ^+^, 1^2^Π, and 2^2^Σ^+^) bear single-reference electron configurations (ESI Table S1). Several state average molecular orbitals, which are plotted by including these four states of ScH^+^ at the CASSCF level, are given in Figure 2. The 1σ, 2σ, 3σ, 1π, and 1δ orbitals chiefly correlate to the 1s of H (with minor ${3d}_{z^{2}}$ and 4s of Sc), ${3d}_{z^{2}}$ (with some 4s) of Sc, 4s of Sc (with a small contribution from 1s of H), 3d_yz_/3d_xz_ of Sc, and $3d_{x^{2}-y^{2}}$/3d_xy_ of Sc, respectively. Based on the contours of the molecular orbitals (Figure 2), the 1σ^2^1δ^1^ electron configuration of 1^2^Δ translates to an approximate ionic Sc^2+^H^−^ structure. According to the NBO population analysis, the 1^2^Δ of ScH^+^ carries Sc^+1.68^H^−0.68^ charge localization with Sc[4s^0.16^3d^1.16^]H[1s^1.66^] electron population. The next three electronic states of ScH^+^ (i.e., 1^2^Σ^+^, 1^2^Π, and 2^2^Σ^+^) bear 1σ^2^2σ^1^, 1σ^2^1π^1^, and 1σ^2^3σ^1^ equilibrium electron configurations with Sc^+1.57^H^−0.57^, Sc^+1.69^H^−0.69^, and Sc^+1.82^H^−0.82^ NBO charge distributions, respectively. Their NBO electron populations are Sc[4s^0.58^3d^0.84^]H[1s^1.55^], Sc[4s^0.19^3d^1.11^]H[1s^1.65^], and Sc[4s^0.47^3d^0.59^]H[1s^1.73^], respectively.

For the sake of textual brevity, from now on, the notations AXZ- [for, aug-cc-pVXZ-DK (of H) cc-pVXZ-DK (of Sc), aug-cc-pVXZ (of H) cc-pVXZ-PP (of Y), aug-cc-pVXZ-DK (of H) cc-pVXZ-DK3 (of La)] and AXZ-C- [for, aug-cc-pVXZ-DK (of H) cc-pwCVXZ-DK (of Sc), aug-cc-pVXZ (of H) cc-pwCVXZ-PP (of Y), aug-cc-pVXZ-DK (of H) cc-pwCVXZ-DK3 (of La)] (X = T, Q, 5) are used throughout the paper to denote the basis set combinations. All our theoretical approaches predicted a 1^2^Δ ground electronic state for the ScH^+^ (Table 2). Recall that according to our A5Z-MRCI potential energy profile (Figure 1), 1^2^Π is slightly more stable than 1^2^Σ^+^. However, A5Z-MRCI calculations built on top of the CASSCF wave functions created by including only 1^2^Δ, 1^2^Σ^+^, 1^2^Π, and 2^2^Σ^+^ states of ScH^+^ predicted 1^2^Σ^+^ to be more stable than 1^2^Π (Table 2). Indeed, all MRCI and MRCI+Q approaches listed in Table 2 predicted 1^2^Σ^+^ to be 369–388 cm^−1^ more stabilized compared to 1^2^Π. Utilization of higher levels of theories such as A5Z-CCSD(T), A5Z-C-CCSD(T), and A5Z-fcFCI decreased the energy difference between 1^2^Σ^+^ and 1^2^Π to ~150 cm^−1^ (Table 2). According to our largest theory that does not account spin–orbit effects, CBS-fcFCI-δcore [i.e., E_δcore_ = E_A5Z-C-CCSD(T)_ − E_A5Z-CCSD(T)_], this energy difference is only 154 cm^−1^ (i.e., the T_e_ values of 1^2^Σ^+^ and 1^2^Π are 1386 and 1540 cm^−1^, respectively). Under all our MRCI and MRCI+Q predictions, the 2^2^Σ^+^ rests at ~15,250 cm^−1^ (Table 2). The core electron correlation is vital for gaining accurate predictions of r_e_ values of the first-row transition metal diatomic species [21,22,23,24]. According to the findings of this study, the 3s^2^3p^6^ electron correlation decreased the r_e_ by 0.03–0.04 Å while simultaneously increasing the ω_e_ by 33–52 cm^−1^ [compare C-CCSD(T) and CCSD(T) r_e_ and ω_e_ values listed in Table 2]. The ω_e_x_e_, B_e_, α_e_, and$\;\bar{D}$_e_ values predicted under different levels for the first four electronic states of ScH^+^ are also given in Table 2.

At the AQZ-MRCI level, ScH^+^(1^2^Δ) carries a D_0_ of 54.46 kcal/mol. This value is in good agreement with the experimental D_0_ of ScH^+^ reported by the Armentrout group (i.e., 55.35 ± 2.31 kcal/mol) [25]. The application of the Davidson correction (i.e., AQZ-MRCI+Q) slightly increased the D_0_ of ScH^+^(1^2^Δ) to 54.54 kcal/mol. The improvement of the basis set from AQZ to A5Z increased the D_0_ by 0.45 kcal/mol for both MRCI and MRCI+Q (Table 2). Both A5Z-CCSD(T) and AQZ-CCSD(T) D_0_ values of ScH^+^(1^2^Δ) are slightly lower compared to the A5Z-MRCI and A5Z-MRCI+Q D_0_ values. As expected, the AQZ-fcFCI and A5Z-fcFCI D_0_ values are closer to the experimental values than the D_0_ of AQZ-CCSD(T) and A5Z-CCSD(T), clearly due to the better representation of electron correlation at fcFCI compared to CCSD(T). The 3s^2^3p^6^ core electron correlation of Sc slightly increased the D_0_ (by ~0.6 kcal/mol). The CBS extrapolation increased the D_0_ by 0.17 kcal/mol compared to the A5Z-fcFCI D_0_ of ScH^+^ (Table 2). The inclusion of the core electron correlation correction (i.e., δcore) to the CBS-fcFCI provided us with a D_0_ of 55.55 kcal/mol (CBS-fcFCI-δcore) for ScH^+^ (1^2^Δ).

**Figure 3:**
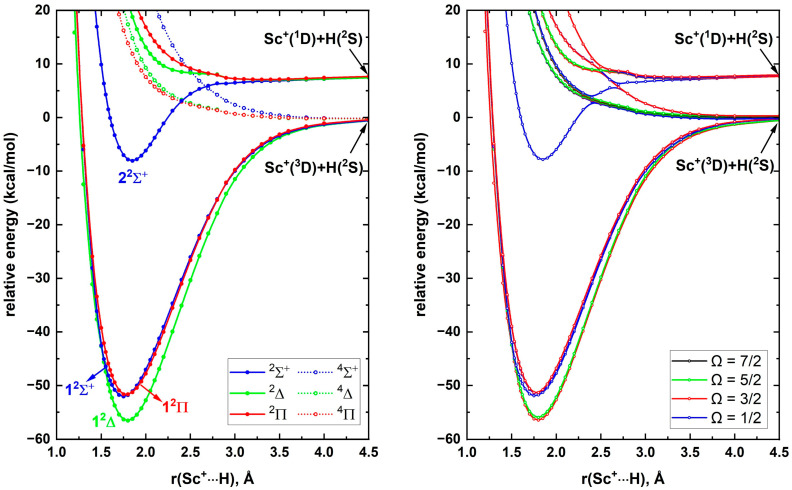
A5Z-C-MRCI spin–free PECs (left panel) and spin–orbit curves (right panel) of ScH^+^ as a function of Sc^+^···H distance [r(Sc^+^···H), Å]. In the left plot the relative energies are referenced to the Sc^+^(^3^D) + H(^2^S) fragments placed at 200 Å, which is set to 0 kcal/mol, whereas, in the right plot the relative energies are with respect to the lowest energy spin–orbit state at 200 Å.

Generally, the spin–orbit coupling effects of the early first-row transition metal-based diatomic species are minor, and similarly we do not expect the spin–orbit coupling of ScH^+^ to be significant. However, full spin–orbit coupling curves are useful for gaining insight on true dissociations of the states of diatomic species and constructing accurate radiative models. Aiming to understand the spin–orbit curves and the corresponding avoided crossings, full spin–orbit curves of ScH^+^ were produced at the A5Z-C-MRCI level and are given in Figure 3 (right panel). Figure 3 (left panel) illustrates the spin–free PECs of ScH^+^ under the same method. Observe that the spin–free A5Z-C-MRCI approach indeed predicted 1^2^Σ^+^ to be more stable than 1^2^Π. The 1^2^Δ, 1^2^Σ^+^, 1^2^Π, and 2^2^Σ^+^ electronic states of ScH^+^ split into Ω = 3/2, 5/2, Ω = 1/2, Ω = 3/2, 1/2, and Ω = 1/2 spin–orbit states of ScH^+^. As expected, the spin–orbit coupling is minor for ScH^+^ at all internuclear distances (compare the spin–orbit curves and parent spin–free PECs of ScH^+^ in Figure 3). The spin–orbit splitting of Sc^+^(^3^D) is minor. Specifically, experimentally the T_e_ of the *J* states of Sc^+^(^3^D) are 0.00, 67.72, and 177.76 cm^−1^, respectively [19]. Hence, the Ω states resulting from the Sc^+^(^3^D)+H(^2^S) nearly degenerate at the bond dissociation limit (Figure 3, right panel). The experimental excitation energy of Sc^+^(^1^D_2_) is 2540.95 cm^−1^. According to our spin–orbit energy profile (Figure 3, right panel) this excitation energy is ~7.9 kcal/mol (or 2763 cm^−1^), which is higher compared to the corresponding experimental value by 222 cm^−1^. This discrepancy could be a result of the size-extensivity issues of MRCI, and the findings may be further improved by the application of MRCI+Q that partially corrects the size-extensivity errors of MRCI. The spin–orbit ground state of ScH^+^ is a 1^2^Δ_3/2_ with a D_0_ of 54.17 kcal/mol (at A5Z-C-MRCI), which is only 0.10 kcal/mol lower than the spin–orbit effect disregarded D_0_ of 1^2^Δ obtained at the same approach. We arrived at our best D_0_ estimate of ScH^+^ by introducing this spin–orbit correction to the CBS-fcFCI-δcore D_0_ of ScH^+^ [i.e., CBS-fcFCI-δcore-δSO]. Indeed, the CBS-fcFCI-δcore-δSO D_0_ of ScH^+^ is in excellent agreement with the experimental value of the Armentrout group (i.e., 55.45 vs. 55.35 ± 2.31 kcal/mol) (Table 3). The Ω = 5/2 component of 1^2^Δ lies 175 cm^−1^ above the 1^2^Δ_3/2_ (ESI Table S2). The next two spin–orbit states of ScH^+^ (Ω = 1/2 at 1582 cm^−1^ and Ω = 1/2 at 1752 cm^−1^) bear 80% 1^2^Σ^+^ + 20% 1^2^Π and 80% 1^2^Π + 20% 1^2^Σ^+^ ΛS compositions, respectively. The proceeding two spin–orbit states of ScH^+^ (Ω = 3/2 at 1779 cm^−1^ and Ω = 1/2 at 17,006 cm^−1^) dominantly correlate to the 1^2^Π and 2^2^Σ^+^ (ESI Table S2). We observed several avoided crossings for the higher energy PECs (ESI Figure S1). The most obvious example is the Ω = 1/2 of 2^2^Σ^+^, which dissociates to the spin–orbit products of Sc^+^(^3^D)+H(^2^S) (Figure 3 right panel and ESI Figure S1). Our final spectroscopic predictions obtained combining the CBS-fcFCI-δcore and MRCI spin–orbit effects [i.e., CBS-fcFCI-δcore-δSO] are listed in Table 3. Specifically, we are reporting T_e_, r_e_, ω_e_, ω_e_x_e_, B_e_, α_e_, $\bar{D}$_e_, and D_0_ values of the first five spin–orbit states of ScH^+^ at the composite CBS-fcFCI-δcore-δSO level to assist future experimental studies of ScH^+^.

**Table 2:** Adiabatic excitation energy (T_e_, cm^−1^), bond length (r_e_, Å), harmonic vibrational frequency (ω_e_, cm^−1^), anharmonicity (ω_e_x_e_, cm^−1^), equilibrium rotational constant (B_e_, cm^−1^), anharmonic correction to the rotational constant (α_e_, cm^−1^), centrifugal distortion constant ($\bar{D}$_e_, cm^−1^), and the bond dissociation energies with respect to the Sc^+^(^3^D; 3d^1^4s^1^)+H(^2^S) fragments (D_0_, kcal/mol) of the first four electronic states of ScH^+^ *^a^*.

State	Method	T_e_	r_e_	ω_e_	ω_e_x_e_	B_e_	α_e_ ×10^−4^	$\bar{D}$_e_ ×10^−6^	D_0_
1^2^Δ	CBS-fcFCI-δcore	…	1.791	1657	22.3	5.333	1074	220	55.55
	CBS-fcFCI	…	1.827	1621	21.1	5.125	1013	205	54.98
	A5Z-fcFCI	…	1.827	1620	21.1	5.122	1013	205	54.81
	AQZ-fcFCI	…	1.828	1619	21.1	5.118	1016	205	54.75
	A5Z-C-CCSD(T)	…	1.791	1657	22.1	5.330	1072	221	55.26
	AQZ-C-CCSD(T)	…	1.793	1653	22.0	5.320	1082	221	54.96
	A5Z-CCSD(T)	…	1.827	1621	21.2	5.122	1019	205	54.69
	AQZ-CCSD(T)	…	1.828	1620	21.3	5.118	1015	204	54.63
	A5Z-MRCI+Q	…	1.826	1617	21.2	5.129	1076	207	54.99
	A5Z-MRCI	…	1.826	1616	21.3	5.128	1022	207	54.91
	AQZ-MRCI+Q	…	1.827	1616	21.3	5.126	1020	206	54.54
	AQZ-MRCI	…	1.827	1616	21.5	5.125	1023	206	54.46
	CCSD(T) [26]	…	1.79						50.10
	MCPF [16]	…	1.830	1568					56.00
	MP2 [26]	…	1.78						43.00
	MCSCF+1+2 [14]	…	1.822	1595					52.7
	Experiment [25]	…							55.35 ± 2.31
1^2^Σ^+^	CBS-fcFCI-δcore	1386	1.745	1648	26.5	5.615	1292	261	51.58
	CBS-fcFCI	1295	1.775	1598	24.6	5.429	1305	251	51.31
	A5Z-fcFCI	1296	1.775	1595	24.5	5.427	1298	251	51.14
	AQZ-fcFCI	1299	1.776	1592	24.4	5.424	1295	251	51.08
	A5Z-C-CCSD(T)	1406	1.745	1648	27.9	5.617	1336	261	51.24
	AQZ-C-CCSD(T)	1334	1.745	1647	26.5	5.614	1299	261	51.18
	A5Z-CCSD(T)	1317	1.774	1597	24.4	5.432	1310	251	50.96
	AQZ-CCSD(T)	1321	1.775	1595	25.1	5.429	1305	252	50.85
	A5Z-MRCI+Q	1026	1.774	1606	32.5	5.433	1832	249	52.06
	A5Z-MRCI	1046	1.775	1595	26.3	5.431	1824	252	51.92
	AQZ-MRCI+Q	1036	1.775	1601	31.9	5.429	1822	250	51.59
	AQZ-MRCI	1056	1.775	1593	30.5	5.426	1814	252	51.45
	MCPF [16]	1700	1.788	1571					
	MCSCF+1+2 [14]		1.776	1532					
1^2^Π	CBS-fcFCI-δcore	1540	1.780	1626	22.5	5.397	1135	238	51.14
	CBS-fcFCI	1432	1.817	1590	22.7	5.180	1073	220	50.93
	A5Z-fcFCI	1439	1.818	1590	22.5	5.176	1074	220	50.74
	AQZ-fcFCI	1447	1.819	1587	22.0	5.171	1077	220	50.66
	A5Z-C-CCSD(T)	1565	1.781	1624	22.3	5.392	1153	238	50.78
	AQZ-C-CCSD(T)	1562	1.783	1622	22.7	5.381	1127	237	50.50
	A5Z-CCSD(T)	1456	1.818	1590	21.7	5.177	1080	220	50.53
	AQZ-CCSD(T)	1465	1.819	1588	21.9	5.171	1075	219	50.49
	A5Z-MRCI+Q	1414	1.816	1587	21.9	5.185	1086	222	51.00
	A5Z-MRCI	1417	1.817	1586	21.5	5.183	1074	221	50.91
	AQZ-MRCI+Q	1423	1.817	1585	21.7	5.180	1071	221	50.53
	AQZ-MRCI	1425	1.817	1584	21.7	5.178	1086	221	50.44
	MCSCF+1+2 [14]		1.816	1560					
2^2^Σ^+^	A5Z-MRCI+Q	15,245	1.861	1555	35.5	4.937	1263	199	11.50
	A5Z-MRCI	15,265	1.861	1554	34.9	4.938	1274	199	11.36
	AQZ-MRCI+Q	15,253	1.862	1551	34.5	4.935	1283	200	11.03
	AQZ-MRCI	15,273	1.861	1554	34.4	4.936	1250	199	10.89

*^a^* The CASSCF wave functions of all MRCI/MRCI+Q calculations were produced by averaging three ^2^A_1_ + one ^2^B_1_ + one ^2^B_2_ + one ^2^A_2_ states. A5Z-C-MRCI and A5Z-C-MRCI+Q spectroscopic constants obtained by state averaging all states given in Figure 3 (left panel) are listed in ESI Table S3.

**Table 3:** CBS-fcFCI-δcore-δSO adiabatic excitation energy (T_e_, cm^−1^), bond length (r_e_, Å), harmonic vibrational frequency (ω_e_, cm^−1^), anharmonicity (ω_e_x_e_, cm^−1^), equilibrium rotational constant (B_e_, cm^−1^), anharmonic correction to the rotational constant (α_e_, cm^−1^), centrifugal distortion constant ($\bar{D}$_e_, cm^−1^), and bond energies (D_0_, kcal/mol) of the first five spin–orbit states of ScH^+^.

State	T_e_	r_e_	ω_e_	ω_e_x_e_	B_e_	α_e_ ×10^−4^	$\bar{D}$_e_ ×10^−6^	D_0_
1^2^Δ_3/2_	…	1.791	1657	23.3	5.323	1047	223	55.45
1^2^Δ_5/2_	175	1.791	1658	23.8	5.324	1025	219	54.95
1^2^Σ^+^_1/2_	1397	1.751	1518	24.6	5.578	1839	249	51.61
1^2^Π_1/2_	1602	1.771	1611	23.8	5.425	1509	240	50.91
1^2^Π_3/2_	1624	1.780	1648	24.9	5.446	1144	235	50.70

### 2.3. YH^+^

The ground state of Y^+^ is a ^1^S with 5s^2^ valence electron configuration in contrast to Sc^+^ which bears a ^3^D (3d^1^4s^1^) ground electronic state [19]. Interestingly, the corresponding ^1^S (4s^2^) state of Sc^+^ is its fourth excited electronic state lying at 11,736.36 cm^−1^. However, the first three excited states of Y^+^ (^3^D; 4d^1^5s^1^, ^1^D; 4d^1^5s^1^, and ^3^F; 4d^2^) and the first three electronic states of Sc^+^ (^3^D; 3d^1^4s^1^, ^1^D; 3d^1^4s^1^, and ^3^F; 3d^2^) are similar [19]. Since the atomic spectra of Sc^+^ and Y^+^ are somewhat different, we expect the electronic spectra of YH^+^ and ScH^+^ to differ as well.

**Figure 4:**
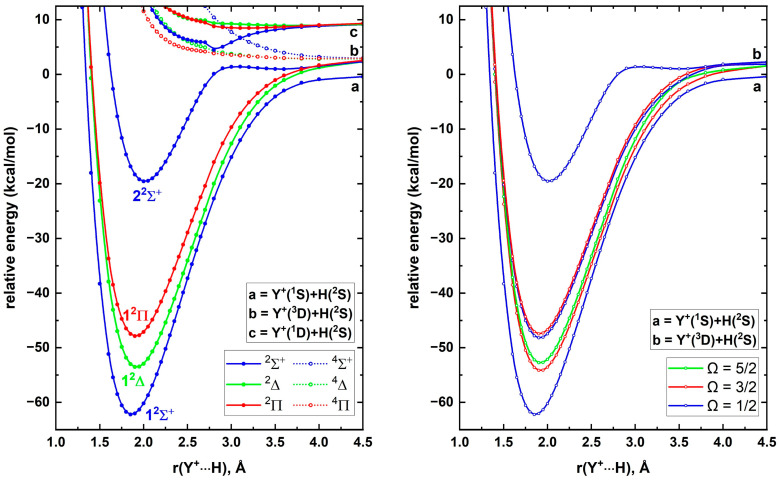
A5Z-C-MRCI spin–free PECs (left panel) and spin–orbit curves (right panel) of YH^+^ as a function of Y^+^···H distance [r(Y^+^···H), Å]. In the left plot the relative energies are referenced to the Y^+^(^1^S) + H(^2^S) fragments placed at 200 Å, which is set to 0 kcal/mol, whereas, in the right plot the relative energies are with respect to the lowest energy spin–orbit state at 200 Å.

For the calculations of YH^+^, the AXZ [aug-cc-pVXZ (of H) cc-pVXZ-PP (of Y)] and AXZ-C [aug-cc-pVXZ (of H) cc-pwCVXZ-PP (of Y)] (X = Q, 5) basis sets were used with the energy-consistent relativistic pseudopotential (ECP28). It should be noted that the utilization of such ECPs for second row-transition metal species is common, especially to avoid convergence issues caused by inner electrons and to minimize the computational cost [27,28,29,30,31,32,33]. To investigate the electronic spectrum of YH^+^, here we have considered the Y^+^(^1^S)+H(^2^S), Y^+^(^3^D)+H(^2^S), and Y^+^(^1^D)+H(^2^S) reactions that produce ^2^Σ^+^, ^4,2^[Σ^+^, Π, Δ], and ^2^[Σ^+^, Π, Δ] molecular states of YH^+^. The A5Z-C-MRCI PECs of these electronic states of YH^+^ are given in Figure 4 (left panel). The ground state of YH^+^ is a 1^2^Σ^+^ originating from the Y^+^(^1^S)+H(^2^S) ground state fragments. All doublet-spin states arising from the Y^+^(^3^D)+H(^2^S) are attractive while its quartet molecular states are repulsive, similar to the PECs of the Sc^+^(^3^D)+H(^2^S) case (Figure 1). The first and second excited states of YH^+^ (i.e., 1^2^Δ and 1^2^Π) smoothly dissociate to Y^+^(^3^D)+H(^2^S). The 2^2^Σ^+^ of the same fragments undergoes an avoided crossing (around 2.6 Å) with the ^2^Σ^+^ PEC coming from the Y^+^(^1^D)+H(^2^S). Recall that the 2^2^Σ^+^ of ScH^+^ originated from the Sc^+^(^1^D; 3d^1^4s^1^)+H(^2^S), which further confirms the true origin (considering the avoided crossing) of YH^+^(2^2^Σ^+^) as Y^+^(^1^D; 4d^1^5s^1^)+H(^2^S). Overall, the first four electronic states of YH^+^ (1^2^Σ^+^, 1^2^Δ, 1^2^Π, and 2^2^Σ^+^) are bound with respect to the ground state fragments similar to the ScH^+^ case. As we expected earlier, the electronic spectra of ScH^+^ and YH^+^ are different, with the first two electronic states of ScH^+^ being 1^2^Δ and 1^2^Σ^+^ while they are opposite in order for YH^+^ (i.e., 1^2^Σ^+^ and 1^2^Δ).

**Figure 5:**
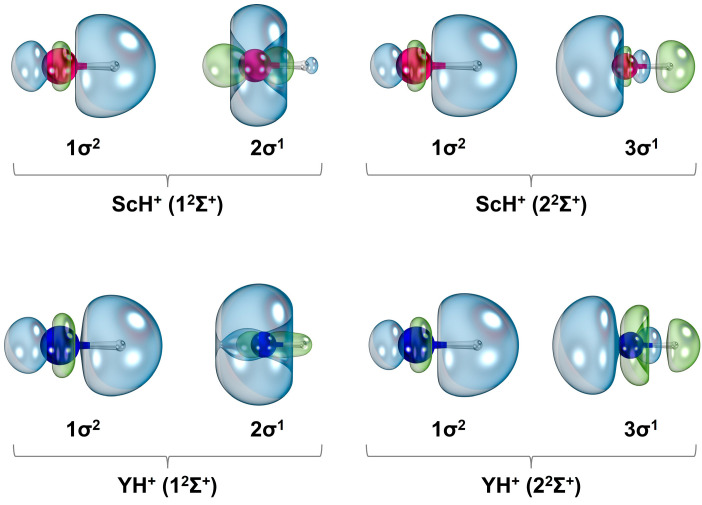
Valence HF molecular orbitals of 1^2^Σ^+^ and 2^2^Σ^+^ states of ScH^+^ and YH^+^. The Sc, Y, and H atoms of each orbital plot are shown in magenta, blue, and gray spheres, respectively. The two phases of orbitals are given in blue and green. A threshold of 90% was used to plot the contours.

The 1^2^Σ^+^, 1^2^Δ, 1^2^Π, and 2^2^Σ^+^ electronic states of YH^+^ bear similar single-reference electron configurations and NBO charges to those of ScH^+^ (i.e., 1^2^Σ^+^: 1σ^2^2σ^1^, 1^2^Δ: 1σ^2^1δ^1^, 1^2^Π: 1σ^2^1π^1^, and 2^2^Σ^+^: 1σ^2^3σ^1^) (ESI Tables S1 and S4). The shapes of the CASSCF state average molecular orbitals resulting from these four states of YH^+^ are qualitatively similar to those of ScH^+^ (Figure 2). The NBO electron populations of 1^2^Δ and 1^2^Π of ScH^+^ and those of the YH^+^ are similar to each other. On the contrary, the electron populations of YH^+^(1^2^Σ^+^, 2^2^Σ^+^) and ScH^+^(1^2^Σ^+^, 2^2^Σ^+^) are slightly different (i.e., Y[5s^0.94^4d^0.43^]H[1s^1.58^] and Sc[4s^0.58^3d^0.84^]H[1s^1.55^] for 1^2^Σ^+^ and Y[5s^0.24^4d^0.79^]H[1s^1.77^] and Sc[4s^0.47^3d^0.59^]H[1s^1.73^] for 2^2^Σ^+^). This substantial difference between the electron population on the Y and Sc atoms is elucidated by their state-specific equilibrium Hartree–Fock (HF) molecular orbitals (Figure 5). Observe that the 2σ of YH^+^(1^2^Σ^+^) is dominated by the 5s of Y, whereas 2σ of ScH^+^(1^2^Σ^+^) is a clear hybrid of ${3d}_{z^{2}}$ and 4s of Sc (Figure 5). On the other hand, the 3σ of ScH^+^(2^2^Σ^+^) bears more s orbital character compared to the s orbital character of the 3σ of YH^+^(2^2^Σ^+^) (Figure 5).

Similar to the ScH^+^ case, several theoretical approaches were utilized to determine the spectroscopic parameters of 1^2^Σ^+^, 1^2^Δ, 1^2^Π, and 2^2^Σ^+^ of YH^+^ (Table 4). Our CBS-fcFCI-δcore estimated that the r_e_, ω_e_, and ω_e_x_e_ of the ground state of YH^+^ are 1.862 Å, 1688 cm^−1^, and 20.5 cm^−1^, respectively. The core electron correlation disregarded calculations predicting that the r_e_ and ω_e_ of 1^2^Σ^+^ are closer to the corresponding MCPF values reported by Pettersson et al. [16]. In all cases, the core electron correlation was found to shorten the r_e_ while increasing the ω_e_ values [compare A5Z-CCSD(T) and A5Z-C-CCSD(T) values in Table 4]. All our core electron correlation accounted D_0_ (of 1^2^Σ^+^) predictions are within the error bars of the experimental D_0_ reported by Sievers et al. (i.e., 61.11 ± 1.84 kcal/mol), which further substantiates the importance of the core electron correlation for gaining accurate predictions of transition metal diatomic species [34]. Our CBS-fcFCI-δcore theoretical approach provided the best D_0_ (i.e., 60.54 kcal/mol), which is only 0.57 kcal/mol larger than the experimental D_0_ of YH^+^ (Table 4) [34].

The spin–orbit states resulting from the first four electronic states of YH^+^ are illustrated in Figure 4 (right panel). There is no spin–orbit splitting for the 1^2^Σ^+^ state of YH^+^. However, the spin–orbit splitting of both 1^2^Δ and 1^2^Π is substantial, especially compared to the splitting of both the 1^2^Δ and 1^2^Π of ScH^+^. Specifically, the Ω = 3/2 and Ω = 5/2 of 1^2^Δ and Ω = 1/2 and Ω = 3/2 of 1^2^Π of YH^+^ are separated by 492 and 263 cm^−1^ (ESI Table S5), while those splits of ScH^+^ are 175 and 27 cm^−1^, respectively. At equilibrium distances, the spin–orbit states of YH^+^ do not carry significant mixings (ESI Table S5), hence their spectroscopic parameters are either same or nearly identical to the spectroscopic parameters of corresponding electronic states (Table 4 and ESI Table S5). Similar to the ScH^+^ case, the CBS-fcFCI-δcore-δSO spectroscopic parameters of YH^+^ are provided for five spin–orbit states (Table 5).

### 2.4. LaH^+^

**Figure 6:**
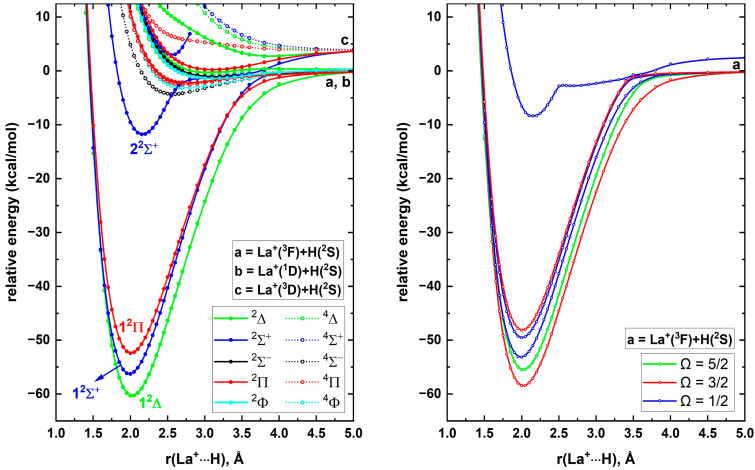
AQZ-C-MRCI spin–free PECs (left panel) and spin–orbit curves (right panel) of LaH^+^ as a function of La^+^···H distance [r(La^+^···H), Å]. In the left plot the relative energies are referenced to the La^+^(^3^F) + H(^2^S) fragments placed at 200 Å, which is set to 0 kcal/mol, whereas, in the right plot the relative energies are with respect to the lowest energy spin–orbit state at 200 Å.

Having obtained a better understanding of the electronic structures of ScH^+^ and YH^+^, next we shifted our attention to LaH^+^. The ground state of La^+^ is a ^3^F with 5d^2^ valence electron configuration. Sc^+^ and Y^+^ populated similar ^3^F(*n*d^2^) in their second and third excited states (at 4802–4988 cm^−1^ and 8003–8744 cm^−1^, respectively) [19]. The first two excited states of La^+^ (i.e., ^1^D; 5d^2^ and ^3^D; 5d^1^6s^1^) lie very close to its ground state (at 1394.46 and 1895.15–3250.35 cm^−1^, respectively) with large spin–orbit splittings [19]. To study LaH^+^, as the first step, the reactions of the first three electronic states of La^+^ with H(^2^S) were studied. The AQZ-C-MRCI PECs of the ^2,4^[Σ^–^, Π, Δ, Φ], ^2^[Σ^+^, Π, Δ], and ^2,4^[Σ^+^, Π, Δ] molecular states originating from La^+^(^3^F)+H(^2^S), La^+^(^1^D)+H(^2^S), and La^+^(^3^D)+H(^2^S) are given in Figure 6 (left panel). The ground electronic state of LaH^+^ is 1^2^Δ, which smoothly dissociates to La^+^(^3^F)+H(^2^S). Similarly, the second excited state of LaH^+^ (i.e., 1^2^Π) is a product of the same fragments. The first excited state of LaH^+^ (i.e., 1^2^Σ^+^) dissociates to La^+^(^1^D)+H(^2^S), but displays an avoided crossing at ~3.75 Å with a ^2^Σ^+^ PEC resulting from La^+^(^3^D)+H(^2^S). Indeed, this ^2^Σ^+^ of La^+^(^3^D)+H(^2^S) turned out to be the third excited state of LaH^+^ (i.e., 2^2^Σ^+^) (Figure 6, left panel). Several doublet and quartet spin PECs of the selected La^+^ + H reactions produce shallow minima that are bound with respect to the ground state fragments. Specifically, the fifth state of LaH^+^ is such a state (i.e., ^4^Σ^-^), with a 2.6 Å r_e_ and a 4.32 kcal/mol binding energy with respect to ground state fragments. We observed another ^2^Σ^+^ state stabilized energetically in between La^+^(^1^D)+H(^2^S) and La^+^(^3^D)+H(^2^S) asymptotes, which are plotted up to 2.8 Å (Figure 6, left panel). Overall, upon considering the first four electronic states, the third and fourth states of all LaH^+^, YH^+^, and ScH^+^ are 1^2^Π and 2^2^Σ^+^. While the ordering of the first two states of LaH^+^ and ScH^+^ is identical (1^2^Δ and 1^2^Σ^+^), the order is swapped in the YH^+^ case to 1^2^Σ^+^ and 1^2^Δ.

The charge distributions and electron populations of 1^2^Δ, 1^2^Σ^+^, 1^2^Π, and 2^2^Σ^+^ of LaH^+^ and YH^+^ are approximately similar to each other (compare the NBO findings listed in ESI Tables S4 and S6). The spectroscopic constants of LaH^+^ calculated under various quantum chemical approaches are given in Table 6. In line with the findings for ScH^+^ and YH^+^, core electron correlation was found to shorten the r_e_ values and increase the ω_e_ values of LaH^+^ (Table 2, Table 4, and Table 6). Our CBS-fcFCI-δcore r_e_, ω_e_, and ω_e_x_e_ values of LaH^+^ are 2.011 Å, 1521 cm^−1^, and 17.0 cm^−1^, respectively. Elkind et al.’s experimentally measured D_0_ of LaH^+^ is 57.19 ± 2.08 kcal/mol [17]. The CBS extrapolation increased the D_0_ of LaH^+^ (1^2^Δ) by 0.51 kcal/mol, which is larger compared to the CBS effect of the ScH^+^ (1^2^Δ) and YH^+^ (1^2^Σ^+^) which are only 0.17 and 0.18 kcal/mol, respectively. Our largest level of theory without the spin–orbit coupling effects (i.e., CBS-fcFCI-δcore) predicted a D_0_ of 64.19 kcal/mol for LaH^+^ (1^2^Δ) with respect to ground state fragments.

Since the spin–orbit coupling effects are dominant for lanthanides, next we evaluated the spin–orbit coupling of LaH^+^. The spin–orbit matrix of LaH^+^ was constructed by including all the electronic states given in the left panel of Figure 6. Due to complexity, only the spin–orbit curves arising from the PECs of the first four electronic states of LaH^+^ are illustrated in the right panel of Figure 6. The ground spin–orbit state of LaH^+^ is an Ω = 3/2 with a dominant 1^2^Δ character (i.e., 98% 1^2^Δ + 2% 1^2^Π) (ESI Table S7). The spin–orbit effect included AQZ-C-MRCI D_0_ of LaH^+^ is 56.36 kcal/mol, which is in excellent agreement with Elkind et al.’s experimental D_0_ [17]. The spin–orbit coupling disregarded AQZ-C-MRCI D_0_ of LaH^+^ is 58.21 kcal/mol, hence we can identify a 1.85 kcal/mol spin–orbit effect for the D_0_ of LaH^+^. If we incorporate this spin–orbit correction into our CBS-fcFCI-δcore D_0_ of LaH^+^ (1^2^Δ) then the D_0_ of LaH^+^ would be 62.34 kcal/mol, which is 3.07 kcal/mol larger than the upper bound of the experimental D_0_ reported by Elkind et al.’s D_0_ (Table 7). Large spin–orbit splittings were observed for the 1^2^Δ (Ω = 3/2 and Ω = 5/2) and 1^2^Π (Ω = 1/2 and Ω = 3/2) of LaH^+^ (Figure 6, right panel and ESI Table S7). Specifically, the aforementioned Ω states of the 1^2^Δ and 1^2^Π are energetically separated by 1042 and 488 cm^−1^, respectively (ESI Table S7). Compared to the corresponding splittings of YH^+^, they are 550 and 225 cm^−1^ larger, respectively. Among the studied Ω states, the third and fourth spin–orbit states of LaH^+^ (i.e., Ω = 1/2 at 1857 cm^−1^ and Ω = 1/2 at 3125 cm^−1^) carry the largest ΛS mixings (i.e., 93% 1^2^Σ^+^ + 7% 1^2^Π and 93% 1^2^Π + 7% 1^2^Σ^+^, respectively).

## 3. Computational Details

All internally contracted MRCI [35,36,37], CCSD(T) [38], and fcFCI [39,40] calculations were executed using the MOLPRO 2023.2 [41,42,43] suite of software. The Davidson relaxed correction (MRCI+Q) [44] implemented in MOLPRO was used to reduce the size extensivity errors of MRCI. The Breit–Pauli Hamiltonian (for Sc, ScH^+^, and LaH^+^) and the spin–orbit pseudopotential operator (for YH^+^) were used for spin–orbit calculations.

Initially, three sets of spin–orbit calculations were performed at the MRCI and MRCI+Q levels of theory to investigate the low-lying states of the Sc atom. For these calculations, CASSCF [45,46,47,48] reference wave functions of three electrons in nine orbitals (CAS[3,9]) and quintuple-ζ quality correlation-consistent basis sets were used. The nine active orbitals are the 4s, five 3d, and three 4p atomic orbitals of the Sc atom. Under the used D_2h_ point group symmetry, these are 3a_g_ (4s, ${3d}_{z^{2}}$, ${3d}_{x^{2}-y^{2}}$), 1b_3u_ (4p_x_), 1b_2u_ (4p_y_), 1b_1g_ (3d_xy_), 1b_1u_ (4p_z_), 1b_2g_ (3d_xz_), and 1b_3g_ (3d_yz_) in symmetry. Specifically, the three sets of calculations of the Sc atom performed are as follows: (1) MRCI/aug-cc-pV5Z-DK (MRCI_Sc_) [49] (2) MRCI/aug-cc-pwCV5Z-DK (C-MRCI_Sc_) [49], and (3) MRCI+Q/aug-cc-pwCV5Z-DK (C-MRCI+Q_Sc_) [49]. In the first case, only the valence electrons were correlated at the MRCI level. In the second and third cases, all valence electrons and 3s^2^3p^6^ core electrons were correlated.

For the calculations of diatomic species, the aug-cc-pVXZ (of H) [50], aug-cc-pVXZ-DK (of H) [50,51], cc-pVXZ-DK (of Sc) [49], cc-pwCVXZ-DK (of Sc) [49], cc-pVXZ-PP (of Y) [52], cc-pwCVXZ-PP (of Y) [52], cc-pVXZ-DK3 (of La) [53], and cc-pwCVXZ-DK3 (of La) [53] basis sets were used (X = T, Q, 5). The Douglas–Kroll basis sets were used with the third-order Douglas–Kroll–Hess Hamiltonian. Upon utilizing the AXZ- basis sets with MRCI, CCSD(T), and fcFCI, only the valence electrons of MH^+^ were correlated. In the AXZ-C- cases, all valence electrons and the core 3s^2^3p^6^ of Sc, 4s^2^4p^6^ of Y, and 5s^2^5p^6^4d^10^ of La were correlated. Note that the 28 inner electrons of Y (1s^2^2s^2^2p^6^3s^2^3p^6^3d^10^) were replaced with ECP28.

The C_2v_ point group symmetry was used for the calculations of diatomic MH^+^. For their MRCI calculations, CASSCF wave functions were provided. Specifically, the CASSCF wave functions of all MH^+^ were produced by placing 3 electrons in 10 orbitals [i.e., CAS(3,10)]. At the bond dissociation limit, these 10 orbitals correspond to the 1s atomic orbital of H atom and (*n*+1)s, five *n*d, and three (*n*+1)p atomic orbitals of Sc, Y, and La (*n* = 3, 4, and 5 for Sc, Y, and La, respectively). Under the applied C_2v_ symmetry, these orbitals are 5a_1_ [1s of H and (*n*+1)s, ${nd}_{z^{2}}$, ${nd}_{x^{2}-y^{2}}$, and (*n*+1)p_z_ of M], 2b_1_ [*n*d_xz_ and (*n*+1)p_x_ of M], 2b_2_ [*n*d_yz_ and (*n*+1)p_y_ of M], and 1a_2_ [*n*d_xy_ of M].

Two MRCI potential energy profiles were constructed for ScH^+^ as a function of the Sc^+^···H distance. The first potential energy profile of ScH^+^ was constructed by including all PECs arising from Sc^+^(^3^D; 3d^1^4s^1^)+H(^2^S), Sc^+^(^1^D; 3d^1^4s^1^)+H(^2^S), Sc^+^(^3^F; 3d^2^)+H(^2^S), and Sc^+^(^1^D; 3d^2^)+H(^2^S) combinations at the A5Z-MRCI. The second MRCI potential energy profile of ScH^+^ was created only for the states resulting from the Sc^+^(^3^D; 3d^1^4s^1^)+H(^2^S) and Sc^+^(^1^D; 3d^1^4s^1^)+H(^2^S) at the A5Z-C-MRCI. At the A5Z-C-MRCI level, the spin–orbit coupling curves of ScH^+^ were also calculated. All electronic states resulting from Sc^+^(^3^D; 3d^1^4s^1^)+H(^2^S) and Sc^+^(^1^D; 3d^1^4s^1^)+H(^2^S) reactions were used to produce its spin–orbit matrix. To introduce the spin–free and spin–orbit potential energy curves of YH^+^ (at A5Z-C-MRCI), all the PECs stemming from Y^+^(^1^S)+H(^2^S), Y^+^(^3^D)+H(^2^S), and Y^+^(^1^D)+H(^2^S) combinations were considered. The spin–free and spin–orbit potential energy curves of LaH^+^ were produced at the AQZ-C-MRCI level by correlating all valence electrons of La and H and 5s^2^5p^6^4d^10^ of La. Here, we have considered all PECs originating from the La^+^(^3^F)+H(^2^S), La^+^(^1^D)+H(^2^S), and La^+^(^3^D)+H(^2^S) fragments.

CCSD(T) and fcFCI calculations constructed on top of the HF wavefunctions were also performed to calculate the PECs (around the equilibrium bond region) of a few low-lying electronic states of MH^+^. For these calculations, aforementioned AXZ- and AXZ-C- basis sets and the corresponding electron correlations were used. The AQZ-fcFCI and A5Z-fcFCI PECs of ScH^+^ and YH^+^ and the ATZ-fcFCI and AQZ-fcFCI PECs of LaH^+^ and their corresponding reference HF PECs were used to extrapolate the PECs to the complete basis set (CBS) limit [i.e., CBS-fcFCI].

The CBS extrapolation of the reference HF energies was carried out according to the scheme introduced by Pansini et al. [54]. Specifically, the static correlation energies were extrapolated to the CBS limit via the following:

$$E_{\infty }^{HF}=\frac{E_{X_{i}}e^{\beta {Χ}_{\iota }}-E_{X_{j}}e^{\beta {Χ}_{j}}}{e^{\beta {Χ}_{i}}-e^{\beta {Χ}_{j}}}$$

where *β* (=1.62) is a universal parameter, and $X_{i}$ and $X_{j}$ are hierarchical numbers related to the cardinal numbers of the basis set [54,55]. The dynamic correlation energies of fcFCI were extrapolated using the unified-single-parameter-extrapolation approach via the following:

$$E_{\infty }^{corr}=E_{X}^{corr}+\frac{\alpha E_{X}^{tot}}{X^{3}}$$

where X is the hierarchical number (4.71 for ScH^+^ and YH^+^ and 3.68 for LaH^+^), and *α* is the fitted parameter at each M^+^-H distance [55,56]. Note that the aforementioned schemes were used for correlating HF and dynamic correlation energies separately since the rate of convergence of HF energy is significantly higher compared to the dynamic correlation energy [55].

To obtain more accurate results for MH^+^, the core electron correlation effects calculated at the CCSD(T) [i.e., E_δcore_ = E_C-CCSD(T)_ − E_CCSD(T)_] were added to the CBS-fcFCI energies to determine the CBS-fcFCI-δcore PECs (i.e., E_CBS-fcFCI-δcore_ = E_CBS-fcFCI_ + E_δcore_). The spin–orbit effects (δSO) calculated at the C-MRCI were introduced to the CBS-fcFCI-δcore PECs to calculate the CBS-fcFCI-δcore-δSO PECs. The MRCI, MRCI+Q, CCSD(T), fcFCI, CBS-fcFCI, CBS-fcFCI-δcore, and CBS-fcFCI-δcore-δSO PECs were used to calculate the r_e_, T_e_, ω_e_, ω_e_x_e_, B_e_, α_e_, and $\bar{D}$_e_ values of the MH^+^. The chemical bonding of the MH^+^ species was further investigated by performing natural bond orbital (NBO) population analysis using the NBO7 [57,58] code linked to MOLPRO.

## 4. Conclusions

In conclusion, the excited states of the Sc atom and potential energy profiles and several spectroscopic parameters of MH^+^ were calculated under various quantum chemical techniques conjoined with correlation-consistent basis sets. First, the excited states of the Sc atom were studied using the MRCI_Sc_, C-MRCI_Sc_, and C-MRCI+Q_Sc_ approaches, and the core electron correlation was found to be vital for their proper description. Next, the full spin–free PECs of MH^+^ were calculated using MRCI theory. All these species bear three strongly attractive 1^2^Δ, 1^2^Σ^+^, and 1^2^Π PECs that are bound with respect to the ground state fragments (by ~46–65 kcal/mol). The ground electronic states of ScH^+^ (1^2^Δ), YH^+^ (1^2^Σ^+^), and LaH^+^ (1^2^Δ) smoothly dissociate to Sc^+^(^3^D)+H(^2^S), Y^+^(^1^S)+H(^2^S), and La^+^(^3^F)+H(^2^S) ground state fragments, respectively. According to the NBO analysis, these electronic states bear Sc^+1.68^H^−0.68^, Y^+1.60^H^−0.60^, and La^+1.64^H^−0.64^ charge localizations and Sc[4s^0.16^3d^1.16^]H[1s^1.66^], Y[5s^0.94^4d^0.43^]H[1s^1.58^], and La[6s^0.05^5d^1.29^]H[1s^1.63^] electron populations, respectively. The first three excited electronic states of ScH^+^ and LaH^+^ are 1^2^Σ^+^, 1^2^Π, and 2^2^Σ^+^ in order, whereas that of YH^+^ are 1^2^Δ, 1^2^Π, and 2^2^Σ^+^. All of the first four electronic states of MH^+^ are dominantly single-reference with 1σ^2^1δ^1^ (1^2^Δ), 1σ^2^2σ^1^ (1^2^Σ^+^), 1σ^2^1π^1^ (1^2^Π), and 1σ^2^3σ^1^ (2^2^Σ^+^) configurations. All these states of MH^+^ can be represented by the approximate M^2+^H^–^ ionic structure.

The spin–orbit coupling effects increased in the order of ScH^+^ < YH^+^ < LaH^+^. The spin–orbit ground states of ScH^+^, YH^+^, and LaH^+^ are 1^2^Δ_3/2_, 1^2^Σ^+^_1/2_, and 1^2^Δ_3/2_, respectively. For these states, our most expensive theoretical approach that addressed the core electron correlations, spin–orbit coupling effects, and CBS extrapolation (i.e., CBS-fcFCI-δcore-δSO) predicted 55.45, 60.54, and 62.34 kcal/mol D_0_ values. Our CBS-fcFCI-δcore-δSO D_0_ values of ScH^+^ and YH^+^ are in excellent agreement with the corresponding experimental D_0_ values reported by the Armentrout group (i.e., 55.35 ± 2.31 kcal/mol [25] and 61.11 ± 1.84 kcal/mol [34]). The D_0_ of LaH^+^ predicted by the CBS-fcFCI-δcore-δSO is 3.07 kcal/mol larger than the upper bound of the experimental D_0_ reported by the Armentrout group (i.e., 57.19 ± 2.08 kcal/mol) [17]. The r_e_, T_e_, ω_e_, ω_e_x_e_, B_e_, α_e_, and $\bar{D}$_e_ values of MH^+^ are also reported. Overall, this work is expected to provide useful information and data for future experimental and theoretical spectroscopic investigations of the titled species, as well as for similar transition metal and lanthanide diatomic species.

## Figures and Tables

**Table 1:** Excitation energies (cm^−1^) of several low-lying states of the Sc atom.

Configuration	Term	*J*	Experiment *^a^*	MRCI_Sc_ *^b^*	C-MRCI_Sc_ *^c^*	C-MRCI+Q_Sc_ *^c^*
[Ar]3d^1^4s^2^	^2^D	3/2	0.0	0	0	0
		5/2	168.3	182	193	221
[Ar]3d^2^4s^1^	^4^F	3/2	11,520.0	14,362	12,216	12,257
		5/2	11,557.7	14,403	12,261	12,304
		7/2	11,610.2	14,459	12,327	12,370
		9/2	11,677.3	14,533	12,416	12,470
[Ar]3d^2^4s^1^	^2^F	5/2	14,926.1	16,904	15,927	15,783
		7/2	15,041.9	17,026	16,065	15,927
[Ar]3d^1^4s^1^4p^1^	^4^F^o^	3/2	15,672.6	14,554	15,364	15,747
		5/2	15,756.5	14,726	15,452	15,835
		7/2	15,881.7	14,785	15,574	15,966
		9/2	16,026.6	14,919	15,721	16,116
[Ar]3d^1^4s^1^4p^1^	^4^D°	1/2	16,009.7	14,791	16,136	16,131
		3/2	16,021.8	14,849	16,185	16,194
		5/2	16,141.0	14,915	16,255	16,258
		7/2	16,210.8	14,993	16,342	16,338
[Ar]3d^1^4s^1^4p^1^	^2^D°	5/2	16,022.7	14,610	15,930	16,081
		3/2	16,096.9	14,729	15,955	16,106

*^a^* Experimental values are from Ref. [19]. *^b^* Only valence electrons of Sc are correlated. *^c^* All valence electrons and 3s^2^3p^6^ core electrons of Sc are correlated.

**Table 4:** Adiabatic excitation energy (T_e_, cm^−1^), bond length (r_e_, Å), harmonic vibrational frequency (ω_e_, cm^−1^), anharmonicity (ω_e_x_e_, cm^−1^), equilibrium rotational constant (B_e_, cm^−1^), anharmonic correction to the rotational constant (α_e_, cm^−1^), centrifugal distortion constant ($\bar{D}$_e_, cm^−1^), and the bond dissociation energies with respect to the Y^+^(^1^S; 5s^2^)+H(^2^S) fragments (D_0_, kcal/mol) of the first four electronic states of YH^+^.

State	Method *^a^*	T_e_	r_e_	ω_e_	ω_e_x_e_	B_e_	α_e_ ×10^−4^	$\bar{D}$_e_ ×10^−6^	D_0_
1^2^Σ^+^	CBS-fcFCI-δcore	…	1.862	1688	20.5	4.881	918	163	60.54
	CBS-fcFCI	…	1.895	1661	20.0	4.713	858	152	58.27
	A5Z-fcFCI	…	1.895	1661	20.1	4.712	858	152	58.09
	AQZ-fcFCI	…	1.895	1661	20.1	4.709	857	152	57.85
	A5Z-C-CCSD(T)	…	1.861	1690	20.4	4.882	918	163	60.25
	A5Z-CCSD(T)	…	1.894	1662	19.9	4.715	857	152	57.98
	A5Z-C-MRCI+Q	…	1.861	1692	20.8	4.885	931	162	60.09
	A5Z-C-MRCI	…	1.860	1690	20.3	4.887	915	164	59.82
	MCPF [16]	…	1.892	1643					59.4
	Experiment [34]	…							61.11 ± 1.84
1^2^Δ	CBS-fcFCI-δcore	3162	1.917	1592	20.0	4.603	878	154	51.53
	CBS-fcFCI	3519	1.954	1561	20.0	4.430	827	143	48.29
	A5Z-fcFCI	3525	1.954	1560	20.0	4.429	829	143	48.10
	AQZ-fcFCI	3530	1.955	1559	20.0	4.425	830	143	47.85
	A5Z-C-CCSD(T)	3162	1.917	1594	20.3	4.603	881	153	51.25
	A5Z-CCSD(T)	3518	1.954	1560	19.3	4.430	828	143	48.00
	A5Z-C-MRCI+Q	3181	1.916	1592	20.2	4.606	895	154	51.05
	A5Z-C-MRCI	3027	1.920	1588	20.2	4.591	844	159	51.22
	MCPF [16]	3058	1.954	1546					
1^2^Π	CBS-fcFCI-δcore	4962	1.905	1573	20.3	4.660	938	164	46.42
	CBS-fcFCI	5085	1.942	1542	20.1	4.487	884	152	43.84
	A5Z-fcFCI	5102	1.942	1540	20.0	4.484	884	152	43.61
	AQZ-fcFCI	5120	1.943	1539	20.0	4.480	886	152	43.32
	A5Z-C-CCSD(T)	4998	1.905	1575	20.3	4.660	938	163	46.03
	A5Z-CCSD(T)	5120	1.942	1542	20.1	4.486	887	152	43.45
	A5Z-C-MRCI+Q	5110	1.904	1565	20.3	4.665	952	164	45.55
	A5Z-C-MRCI	5014	1.910	1564	20.9	4.637	958	164	45.56
2^2^Σ^+^	A5Z-C-MRCI+Q	15,226	2.010	1415	20.2	4.188	950	147	16.71
	A5Z-C-MRCI	14,914	2.015	1414	20.4	4.168	941	144	17.33

*^a^* The CASSCF wave functions of the A5Z-C-MRCI/A5Z-C-MRCI+Q calculations were produced by averaging all states given in Figure 4 (left panel).

**Table 5:** CBS-fcFCI-δcore-δSO adiabatic excitation energy (T_e_, cm^−1^), bond length (r_e_, Å), harmonic vibrational frequency (ω_e_, cm^−1^), anharmonicity (ω_e_x_e_, cm^−1^), equilibrium rotational constant (B_e_, cm^−1^), anharmonic correction to the rotational constant (α_e_, cm^−1^), centrifugal distortion constant ($\bar{D}$_e_, cm^−1^), and bond energies (D_0_, kcal/mol) of the first five spin–orbit states of YH^+^.

State	T_e_	r_e_	ω_e_	ω_e_x_e_	B_e_	α_e_ ×10^−4^	$\bar{D}$_e_ ×10^−6^	D_0_
1^2^Σ^+^_1/2_	…	1.862	1688	20.5	4.882	918	163	60.54
1^2^Δ_3/2_	2948	1.917	1592	20.1	4.602	880	154	52.24
1^2^Δ_5/2_	3440	1.917	1584	20.1	4.597	880	155	50.83
1^2^Π_1/2_	4849	1.906	1573	20.4	4.658	940	163	46.85
1^2^Π_3/2_	5111	1.905	1572	20.5	4.661	938	164	46.10

**Table 6:** Adiabatic excitation energy (T_e_, cm^−1^), bond length (r_e_, Å), harmonic vibrational frequency (ω_e_, cm^−1^), anharmonicity (ω_e_x_e_, cm^−1^), equilibrium rotational constant (B_e_, cm^−1^), anharmonic correction to the rotational constant (α_e_, cm^−1^), centrifugal distortion constant ($\bar{D}$_e_, cm^−1^), and the bond dissociation energies with respect to the La^+^(^3^F; 5d^2^)+H(^2^S) fragments (D_0_, kcal/mol) of the first four electronic states of LaH^+^.

State	Method *^a^*	T_e_	r_e_	ω_e_	ω_e_x_e_	B_e_	α_e_ ×10^−4^	$\bar{D}$_e_ ×10^−6^	D_0_
1^2^Δ	CBS-fcFCI-δcore	…	2.011	1521	17.0	4.166	706	125	64.19
	CBS-fcFCI	…	2.064	1479	15.9	3.956	650	113	64.65
	AQZ-fcFCI	…	2.063	1479	16.1	3.957	645	113	64.14
	ATZ-fcFCI	…	2.063	1478	16.1	3.958	632	114	63.71
	AQZ-C-CCSD(T)	…	2.010	1522	16.9	4.169	693	125	63.55
	AQZ-CCSD(T)	…	2.063	1482	16.5	3.959	649	114	64.02
	AQZ-C-MRCI+Q	…	2.013	1516	15.6	4.160	882	126	61.88
	AQZ-C-MRCI	…	2.022	1499	14.9	4.122	777	128	58.21
	Experiment [17]								57.19 ± 2.08
1^2^Σ^+^	CBS-fcFCI-δcore	1363	1.975	1558	17.9	4.320	745	133	60.24
	CBS-fcFCI	1302	2.026	1524	17.8	4.107	703	119	60.93
	AQZ-fcFCI	1298	2.025	1523	17.8	4.110	692	119	60.43
	ATZ-fcFCI	1282	2.024	1521	17.7	4.113	720	121	60.04
	AQZ-C-CCSD(T)	1364	1.973	1558	17.9	4.326	759	133	59.60
	AQZ-CCSD(T)	1306	2.024	1524	17.7	4.112	728	119	60.29
	AQZ-C-MRCI+Q	1381	1.977	1556	18.1	4.312	569	135	57.94
	AQZ-C-MRCI	1421	1.987	1542	18.7	4.268	683	133	54.12
1^2^Π	CBS-fcFCI-δcore	1937	1.991	1512	17.3	4.251	722	134	58.66
	CBS-fcFCI	1702	2.045	1472	16.4	4.030	682	120	59.85
	AQZ-fcFCI	1737	2.044	1471	16.3	4.031	664	121	59.24
	ATZ-fcFCI	1771	2.044	1467	16.3	4.031	693	122	58.72
	AQZ-C-CCSD(T)	2003	1.989	1513	16.7	4.254	707	134	57.83
	AQZ-CCSD(T)	1767	2.044	1473	16.2	4.034	673	121	59.04
	AQZ-C-MRCI+Q	2417	1.993	1518	17.0	4.243	733	128	55.04
	AQZ-C-MRCI	2771	2.006	1497	18.0	4.185	735	136	50.36
2^2^Σ^+^	AQZ-C-MRCI+Q	16,882	2.144	1385	26.1	3.666	480	114	13.76
	AQZ-C-MRCI	16,982	2.163	1352	28.0	3.599	482	109	9.81

*^a^* The CASSCF wave functions of the AQZ-C-MRCI/AQZ-C-MRCI+Q calculations were produced by averaging all states given in Figure 6 (left panel).

**Table 7:** CBS-fcFCI-δcore-δSO adiabatic excitation energy (T_e_, cm^−1^), bond length (r_e_, Å), harmonic vibrational frequency (ω_e_, cm^−1^), anharmonicity (ω_e_x_e_, cm^−1^), equilibrium rotational constant (B_e_, cm^−1^), anharmonic correction to the rotational constant (α_e_, cm^−1^), centrifugal distortion constant ($\bar{D}$_e_, cm^−1^), and bond energies (D_0_, kcal/mol) of the first five spin–orbit states of LaH^+^.

Ω	T_e_	r_e_	ω_e_	ω_e_x_e_	B_e_	α_e_ ×10^−4^	$\bar{D}$_e_ ×10^−6^	D_0_
1^2^Δ_3/2_	0	2.010	1520	17.0	4.168	758	124	62.34
1^2^Δ_5/2_	1041	2.010	1522	16.8	4.169	709	127	59.36
1^2^Σ^+^_1/2_	1800	1.977	1548	18.1	4.309	734	134	57.08
1^2^Π_1/2_	2289	1.989	1516	15.7	4.256	728	134	55.80
1^2^Π_3/2_	2779	1.990	1510	17.9	4.250	750	134	54.40

## Data Availability

The original contributions presented in this study are included in the article/Supplementary Material. Further inquiries can be directed to the corresponding author.

## Supplementary Materials

The following supporting information can be downloaded at: https://www.mdpi.com/article/10.3390/molecules30112435/s1, Table S1: Electron configurations and NBO findings of ScH^+^; Table S2: Spectroscopic constants and ΛS compositions of spin-orbit states of ScH^+^; Figure S1: A5Z-C-MRCI spin-orbit curves of ScH^+^; Table S3: T_e_, r_e_, ω_e_, and ω_e_x_e_ of ScH^+^ at A5Z-C-MRCI and A5Z-C-MRCI+Q; Table S4: Electron configurations and NBO findings of YH^+^; Table S5: Spectroscopic constants and ΛS compositions of spin-orbit states of YH^+^; Table S6: Electron configurations and NBO findings of LaH^+^; Table S7: Spectroscopic constants and ΛS compositions of spin-orbit states of LaH^+^; Table S8: Upper bounds of the CBS and δcore uncertainties of r_e_ and D_0_ of MH^+^.
