# Supplementary material for: An Ab Initio Electronic Structure Investigation of the Ground and Excited States of ScH+, YH+, and LaH+
Source: Molecules. 2025 Jun 2;30(11):2435. doi: 10.3390/molecules30112435 (PMC12156933; doi:10.3390/molecules30112435)
Supplement: Supplementary file 1 [file molecules-30-02435-s001.zip › molecules-3627257-supplementary.pdf]

Electronic Supplementary Information

An *Ab Initio* Electronic Structure Investigation of the Ground and Excited States of ScH^+, YH^+, and LaH^+

Isuru R. Ariyarathna

Theoretical Division, Los Alamos National Laboratory, Los Alamos, NM 87545, United States

E-mail: isuru@lanl.gov

Contents

Table S1 Electron configurations and NBO findings of ScH^+ Page S2
Table S2 Spectroscopic constants ΛS compositions of spin-orbit states of ScH^+ Page S2
Figure S1 A5Z-C-MRCI spin-orbit curves of ScH^+ Page S2
Table S3 T_e, r_e, ω_e, and $\omega_e x_e$ of ScH^+ at A5Z-C-MRCI and A5Z-C-MRCI+Q Page S3
Table S4 Electron configurations and NBO findings of YH^+ Page S3
Table S5 Spectroscopic constants ΛS compositions of spin-orbit states of YH^+ Page S3
Table S6 Electron configurations and NBO findings of LaH^+ Page S4
Table S7 Spectroscopic constants ΛS compositions of spin-orbit states of LaH^+ Page S4
Table S8 Upper bounds of the CBS and δcore uncertainties of r_e and D_0 of MH^+ Page S4

Table S1. Dominant electron configurations of the first 4 electronic states of ScH^+. NBO charges and populations were calculated at A5Z-HF.

State [a]	Coefficient	Configuration	NBO Charge	NBO Population
$1^2\Delta$	0.99	$1\sigma^2 1\delta^1$	$Sc^{+1.68}H^{-0.68}$	$Sc[4s^{0.16}3d^{1.16}]H[1s^{1.66}]$
$1^2\Sigma^+$	0.95	$1\sigma^2 2\sigma^1$	$Sc^{+1.57}H^{-0.57}$	$Sc[4s^{0.58}3d^{0.84}]H[1s^{1.55}]$
$1^2\Pi$	0.98	$1\sigma^2 1\pi^1$	$Sc^{+1.69}H^{-0.69}$	$Sc[4s^{0.19}3d^{1.11}]H[1s^{1.65}]$
$2^2\Sigma^+$	0.92	$1\sigma^2 3\sigma^1$	$Sc^{+1.82}H^{-0.82}$	$Sc[4s^{0.47}3d^{0.59}]H[1s^{1.73}]$

Table S2. Excitation energy (T_e, cm^{-1}), bond length (r_e, Å), harmonic vibrational frequency (ω_e, cm^{-1}), anharmonicity ($\omega_e x_e$, cm^{-1}), equilibrium rotational constant (B_e, cm^{-1}), anharmonic correction to the rotational constant (α_e, cm^{-1}), centrifugal distortion constant ($\bar{D}_e$, cm^{-1}), bond dissociation energies (D_0, kcal/mol), and % ΛS composition of a few low-lying spin-orbit states of ScH^+ at A5Z-C-MRCI level.

Ω	T_e	r_e	ω_e	$\omega_e x_e$	B_e	α_e x10^{-4}	$\bar{D}_e$ x10^{-6}	D_0	ΛS composition
3/2	...	1.794	1651	23.6	5.218	1158	218	54.17	100% $1^2\Delta$
5/2	175	1.794	1645	24.0	5.215	1129	219	53.62	100% $1^2\Delta$
1/2	1582	1.755	1572	17.6	5.549	1987	241	49.75	80% $1^2\Sigma^+$ + 20% $1^2\Pi$
1/2	1752	1.765	1670	23.1	5.486	1456	244	49.15	80% $1^2\Pi$ + 20% $1^2\Sigma^+$
3/2	1779	1.775	1679	27.6	5.430	1145	221	48.92	100% $1^2\Pi$
1/2	17006	1.846	1524	38.6	5.486	1397	238	5.66	100% $2^2\Sigma^+$

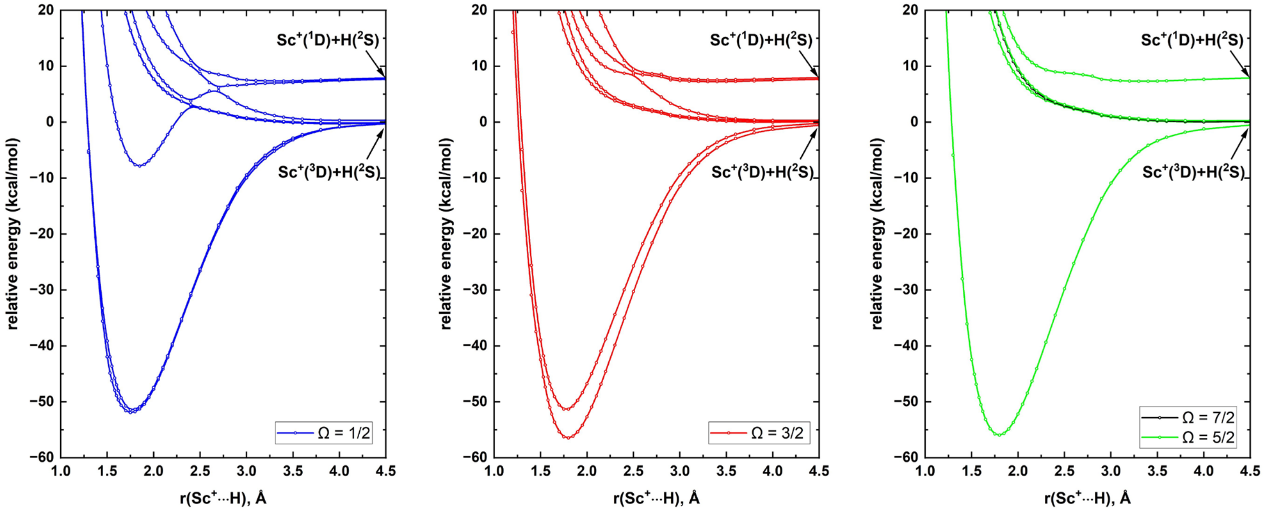

Figure S1. A5Z-C-MRCI spin-orbit curves of ScH^+.

Table S3. A5Z-C-MRCI and A5Z-C-MRCI+Q adiabatic excitation energy (T_e, cm^{-1}), bond length (r_e, Å), harmonic vibrational frequency (ω_e, cm^{-1}), and anharmonicity ($\omega_e x_e$, cm^{-1}) of the first four electronic states of ScH^+.

State	Method[a]	T_e	r_e	ω_e	$\omega_e x_e$	D_0
$1^2\Delta$	A5Z-C-MRCI+Q	...	1.789	1632	24.1	56.64
	A5Z-C-MRCI	...	1.794	1649	23.7	56.57
$1^2\Sigma^+$	A5Z-C-MRCI+Q	1231	1.745	1618	27.3	
	A5Z-C-MRCI	1574	1.750	1608	30.4	
$1^2\Pi$	A5Z-C-MRCI+Q	1673	1.768	1591	22.6	
	A5Z-C-MRCI	1695	1.773	1587	22.7	
$2^2\Sigma^+$	A5Z-C-MRCI+Q	16370	1.843	1490	41.1	
	A5Z-C-MRCI	16960	1.846	1519	40.2	

[a] The CASSCF wave functions of the MRCI/MRCI+Q calculations were produced by state averaging all states given in Figure 3 (left panel).

Table S4. Dominant electronic configurations of the first 4 electronic states of YH^+. NBO charges and populations were calculated at A5Z-HF.

State[a]	Coefficient	Configuration	NBO Charge	NBO Population
$1^2\Sigma^+$	0.96	$1\sigma^2 2\sigma^1$	$Y^{+1.60}H^{-0.60}$	$Y[5s^{0.94}4d^{0.43}]H[1s^{1.58}]$
$1^2\Delta$	0.97	$1\sigma^2 1\delta^1$	$Y^{+1.68}H^{-0.68}$	$Y[5s^{0.12}4d^{1.20}]H[1s^{1.66}]$
$1^2\Pi$	0.96	$1\sigma^2 1\pi^1$	$Y^{+1.69}H^{-0.69}$	$Y[5s^{0.14}4d^{1.15}]H[1s^{1.66}]$
$2^2\Sigma^+$	0.96	$1\sigma^2 3\sigma^1$	$Y^{+1.86}H^{-0.86}$	$Y[5s^{0.24}4d^{0.79}]H[1s^{1.77}]$

Table S5. Excitation energy (T_e, cm^{-1}), bond length (r_e, Å), harmonic vibrational frequency (ω_e, cm^{-1}), anharmonicity ($\omega_e x_e$, cm^{-1}), equilibrium rotational constant (B_e, cm^{-1}), anharmonic correction to the rotational constant (α_e, cm^{-1}), centrifugal distortion constant ($\bar{D}_e$, cm^{-1}), and % ΛS composition of several low-lying spin-orbit states of YH^+ at A5Z-C-MRCI level.

Ω	T_e	r_e	ω_e	$\omega_e x_e$	B_e	α_e x10^{-4}	$\bar{D}_e$ x10^{-6}	ΛS composition
1/2	...	1.860	1688	20.4	4.887	918	164	100% $1^2\Sigma^+$
3/2	2812	1.920	1582	20.1	4.590	881	154	99% $1^2\Delta$ + 1% $1^2\Pi$
5/2	3304	1.919	1571	20.1	4.592	880	155	100% $1^2\Delta$
1/2	4901	1.910	1561	20.8	4.635	950	166	100% $1^2\Pi$
3/2	5164	1.910	1562	21.0	4.638	947	164	99% $1^2\Pi$ + 1% $1^2\Delta$
1/2	14925	2.014	1415	20.4	4.172	956	145	100% $2^2\Sigma^+$

Table S6. Dominant electronic configurations of the first 4 electronic states of LaH^+. NBO charges and populations were calculated at AQZ-HF.

State [a]	Coefficient	Configuration	NBO Charge	NBO Population
$1^2\Delta$	0.96	$1\sigma^2 1\delta^1$	$La^{+1.64}H^{-0.64}$	$La[6s^{0.05}5d^{1.29}]H[1s^{1.63}]$
$1^2\Sigma^+$	0.96	$1\sigma^2 2\sigma^1$	$La^{+1.59}H^{-0.59}$	$La[6s^{0.84}5d^{0.54}]H[1s^{1.58}]$
$1^2\Pi$	0.95	$1\sigma^2 1\pi^1$	$La^{+1.65}H^{-0.65}$	$La[6s^{0.06}5d^{1.25}]H[1s^{1.64}]$
$2^2\Sigma^+$	0.94	$1\sigma^2 3\sigma^1$	$La^{+1.86}H^{-0.86}$	$La[6s^{0.27}5d^{0.71}]H[1s^{1.77}]$

Table S7. Excitation energy (T_e, cm^{-1}), bond length (r_e, Å), harmonic vibrational frequency (ω_e, cm^{-1}), anharmonicity ($\omega_e x_e$, cm^{-1}), equilibrium rotational constant (B_e, cm^{-1}), anharmonic correction to the rotational constant (α_e, cm^{-1}), centrifugal distortion constant ($\bar{D}_e$, cm^{-1}), and % ΛS composition of several low-lying spin-orbit states of LaH^+ at AQZ-C-MRCI level.

Ω	T_e	r_e	ω_e	$\omega_e x_e$	B_e	α_e x10^{-4}	$\bar{D}_e$ x10^{-6}	ΛS composition
3/2	...	2.021	1498	14.7	4.141	793	123	98% $1^2\Delta$ + 2% $1^2\Pi$
5/2	1042	2.021	1500	14.4	4.154	773	124	100% $1^2\Delta$
1/2	1857	1.989	1532	19.3	4.275	721	135	93% $1^2\Sigma^+$ + 7% $1^2\Pi$
1/2	3125	2.004	1498	17.9	4.210	728	133	93% $1^2\Pi$ + 7% $1^2\Sigma^+$
3/2	3613	2.006	1494	20.3	4.204	754	132	98% $1^2\Pi$ + 2% $1^2\Delta$
1/2	17524	2.131	1310	16.6	3.726	1737	111	98% $2^2\Sigma^+$

Table S8. Upper bounds of the CBS uncertainties and the core electron correlations of MH^+ species.

Species	Upper bound of the CBS uncertainty		Upper bound of the core correlation uncertainty	
	r_e	D_0	r_e	D_0
LaH^+ ($1^2\Delta$)	0.001	0.51	-0.053	-0.46
YH^+ ($1^2\Sigma^+$)	0.000	0.18	-0.033	2.27
ScH^+ ($1^2\Delta$)	0.000	0.17	-0.036	0.57

The upper bounds of the CBS uncertainties and the core electron correlation effects were estimated for the r_e and D_0 values of the ground states of MH^+ species. Specifically, we can expect the upper bound of the CBS uncertainty of $r_e = r_{e\ [CBS\text{-}fcFCI]} - r_{e\ [AXZ\text{-}fcFCI]}$, upper bound of the CBS uncertainty of $D_0 = D_{0\ [CBS\text{-}fcFCI]} - D_{0\ [AXZ\text{-}fcFCI]}$, upper bound of the core electron correlation uncertainty of $r_e = r_{e\ [CBS\text{-}fcFCI\text{-}\delta core]} - r_{e\ [CBS\text{-}fcFCI]}$, and upper bound of the core electron correlation uncertainty of $D_0 = D_{0\ [CBS\text{-}fcFCI\text{-}\delta core]} - D_{0\ [CBS\text{-}fcFCI]}$. X = Q for LaH^+ and X = 5 for ScH^+ and YH^+. Overall, based on the ground state r_e and D_0 values (in Table S8) the core electron correlation effects are significant compared to the CBS correction.
